# Melatonin deficiency decreases brown adipose tissue acute thermogenic capacity of in rats measured by ^18^F-FDG PET

**DOI:** 10.1186/s13098-020-00589-1

**Published:** 2020-09-21

**Authors:** Bruno Halpern, Marcio C. Mancini, Caroline Mendes, Camila Maria Longo Machado, Silvana Prando, Marcelo Tatit Sapienza, Carlos Alberto Buchpiguel, Fernanda Gaspar do Amaral, José Cipolla-Neto

**Affiliations:** 1 Department of Endocrinology and Metabolism, Hospital das Clinicas da Faculdade de Medicina de São Paulo, São Paulo, Brazil; 2 Department of Physiology and Biophysics, Institute of Biomedical Sciences, Universidade de São Paulo, São Paulo, Brazil; 3 Nuclear Medicine Institute, Hospital das Clinicas da Faculdade de Medicina de São Paulo, São Paulo, Brazil; 4 Department of Physiology, Federal University of São Paulo, São Paulo, Brazil

**Keywords:** Brown adipose tissue, Melatonin, Obesity, Circadian rhythms, FDG-PET, Thermogenesis, UCP-1

## Abstract

**Objective:**

Melatonin has been shown to increase brown adipose tissue (BAT) mass, which can lead to important metabolic effects, such as bodyweight reduction and glycemic improvement. However, BAT mass can only be measured invasively and. The gold standard for non-invasive measurement of BAT activity is positron emission tomography with 2-deoxy-2-[fluorine-18] fluoro-d-glucose (^18^F-FDG PET). There is no study, to our knowledge, that has evaluated if melatonin influences BAT activity, measured by this imaging technique in animals.

**Methods:**

Three experimental groups of Wistar rats (control, pinealectomy, and pinealectomy replaced with melatonin) had an ^18^F-FDG PET performed at room temperature and after acute cold exposure. The ratio of increased BAT activity after cold exposure/room temperature was called “acute thermogenic capacity” (ATC) We also measured UCP-1 mRNA expression to correlate with the ^18^F-FDG PET results.

**Results:**

Pinealectomy led to reduced acute thermogenic capacity, compared with the other groups, as well as reduced UCP1 mRNA expression.

**Conclusion:**

Melatonin deficiency impairs BAT response when exposed to acute cold exposure. These results can lead to future studies of the influence of melatonin on BAT, in animals and humans, without needing an invasive evaluation of BAT.

## Background

Melatonin is a pineal hormone, produced at night, which has a critical role in the synchronization of circadian rhythms, with known metabolic effects in many animal species [1, 2]. One of its metabolic effects in rodents is a reduction in body weight with a minimal decrease in food intake, suggesting an action on energy expenditure, which possibly relates to activation of brown adipose tissue (BAT) [1–5]. Indeed, many experimental models have shown the role of melatonin on BAT recruitment [3, 6].

BAT is a thermogenic tissue, whose primary function is thermoregulation via non-shivering thermogenesis [7]. Thermogenesis occurs due to a unique and specific enzyme called uncoupling protein-1 (UCP-1), which uncouples ATP energy production in the mitochondria, generating heat [8]. Brown adipose tissue is activated by sympathetic noradrenergic stimuli, and cold is the most important physiological stimulus known [7, 8].

Recently, BAT research has increased after positron emission tomography with 2-deoxy-2-[fluorine-18] fluoro-d-glucose (^18^F-FDG PET) has shown that humans still possess active BAT, especially after cold exposure [9, 10]. In this context, a lack of BAT activation could have a potential role in weight gain in humans. Therefore, targeting BAT activation could be promising for the treatment of obesity and/or type 2 diabetes [11].

Many compounds have been studied to determine the ability of BAT recruitment and activation, including melatonin [12, 13]. However, in the literature, several melatonin studies conducted in animals measured BAT mass, in order to analyze BAT recruitment and activation, and some of them focused on BAT thermogenesis, measured by calorimetry or cytochrome activity [3], yet none of them used 18F-FDG PET, which is the gold standard for BAT measurement in humans. More recently, some studies have evaluated a possible increase in BAT and in thermoregulatory responses, using infrared thermography [5, 14, 15].

It is worth highlighting that an increase in BAT mass (recruitment) does not necessarily lead to an increased thermogenic response (activation), if there is no physiological need for heat production [7, 8, 16]. In this regard, an increase in BAT mass or UCP-1 expression would not necessarily mean an increase in BAT uptake in ^18^F-FDG PET [16, 17]. Further concerning this point, different protocols of cold exposure or other adrenergic stimuli could lead to different patterns of BAT uptake by ^18^F-FDG PET that do not necessarily reflect the total BAT mass of an individual [16].

As ^18^F-FDG PET is the gold-standard method for the detection of BAT in humans [18], it would be relevant to use this methodology in experimental studies to understand the role of melatonin deficiency and its supplementation on BAT thermogenic responses. This would be important, not only for melatonin, but also for any other compound that could potentially increase BAT mass (since an increase in BAT mass in humans, by any compound, if not increase thermogenesis as well, could only be detected by tissue biopsy and would probably have minimal therapeutic implications).

In the present study, we ought to determine if melatonin deficiency in experimental models of pinealectomized Wistar rats decreases BAT activation, evaluated by Positron emission tomography-computed tomography (PET-CT), at room temperature and after cold exposure, and if melatonin replacement could revert these changes.

## Method

### Animals and groups

Young male Wistar rats (3 months old) were maintained under a 12-h light/12-h dark cycle, 23 ± 2 °C at the animal facility room of the Neurobiology Lab in the Department of Physiology and Biophysics at the University of São Paulo. All the experimental procedures followed the animal welfare guides within the protocols approved by the Institute of Biomedical Sciences Ethics Committee of the University of São Paulo, under number 30460114.5.3001.5467.

For the PET-CT analysis, the animals were assigned to three groups: healthy animals with an intact pineal gland (C, n = 6); pinealectomized (PINX) animals (P, n = 7); and PINX rats being treated with melatonin (PM, n = 6) from the first day of surgery. A pinealectomy was performed 1 month before the experiment (animals were 2-months old), as previously described [19], and melatonin (1.0 mg/kg of body weight) (Sigma‐Aldrich, St. Louis, MO, USA) was added to their drinking water, exclusively during the dark phase. The concentration in the drinking water solution was corrected daily, using the ingested volume from the previous night. This melatonin protocol was done to mimic physiological levels of melatonin and have already been used in several of our group’s experiments (peak melatonin plasmatic level of 150 pg/ml) [5, 6, 20].

For the UCP1 mRNA expression, we used a larger number of male Wistar rats (15C, 10 P, and 12PM) that, despite not being the same animals that performed the PET-CT analysis, received the exact same protocol of PINX and melatonin supplementation.

#### Positron emission tomography-computed tomography (PET-CT)

Briefly a small animal imaging Positron emission tomography coupled to a computerized tomography machine was used to follow 2-deoxy-2-[fluorine-18]fluoro-d-glucose physiological uptake in Rats (Additional file 1: Figure S1). Basal imaging (pre-thermogenic stimuli) and 2-days image (post-thermogenic stimuli) were performed using 18F-FDG PET/CT to determine the 18F-FDG uptake images were performed twice, with an interval of 2 days, in both room temperature (23 ± 2 °C) and after an acute cold challenge. During the acute cold challenge, rats were exposed to the environmental temperature of 4 ± 2 °C for 4 h, and immediately afterward, they were scanned using 18F-FDG PET/CT. Our freezing protocol was performed according to the literature available at that moment [21]. The images were performed around ZT 6 (6 h after lights on) to preserve the BAT peak activity in rats as described before [22].

**Fig. 1:**
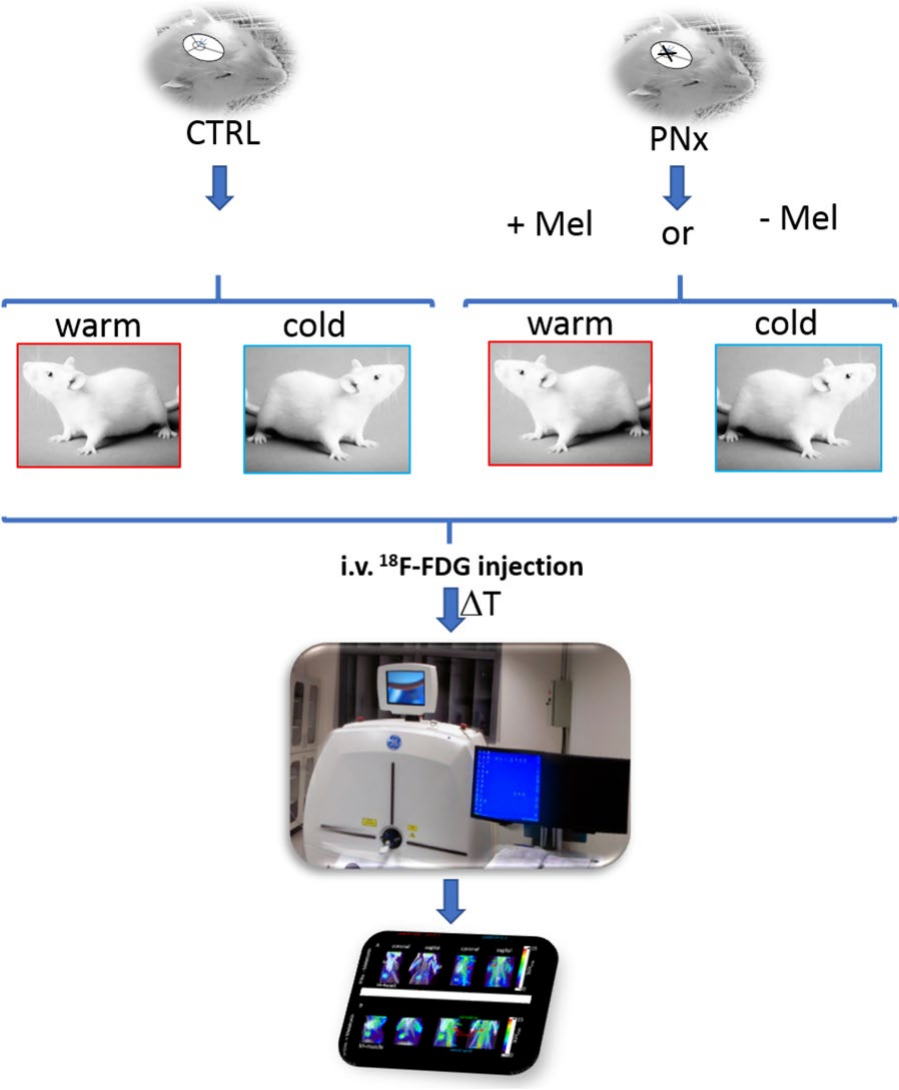
Schematic representation of the experimental imaging procedure

The animals received i.v. 2.474 mCi (91.54 MBq) of 18F-FDG and images were obtained by small imaging PET/CT (Triumph II trimodality System, Gamma Medica/Trifoil imaging), with a field of view (FOV) of 80 mm, matrix 240 × 240. Images were then co-registered with CT for anatomic correlation and analyzed by at least 2 nuclear medicine specialists, using a manually drawed region of interest (ROI) in the interscapular area. The ROI was used to evaluate the interscapular maximal Standard Value Uptake (Max SUV). We analyzed data from both experiments separately as well as the ratio of individual SUV increase (maximal SUV after a cold challenge/maximal SUV in room temperature),which was interpreted as an “acute thermogenic activity of BAT.” The BAT acute thermogenic capacity was measured in each animal individually and the mean results of each group was then compared. The schematic representation of the intervention is illustrated in Fig. 1.

### UCP-1 mRNA expression

Different male Wistar rats, that received the same protocol of those who performed the imaging technique were euthanized at ZT18 (6 h after lights off) and interscapular brown adipose tissue was collected for UCP-1 mRNA expression analysis. BAT was maintained in dry ice and homogenized in 1 ml of Trizol Reagent (Invitrogen, Carlsbad, CA, USA) for total RNA extraction according to the manufacturer’s instructions. Samples were then treated with DNase (Turbo DNA-free™, Ambion, Austin, TX, USA) and total RNA was quantified at 260 nm using a spectrophotometer, with acceptable 260/280 ratios of 1.8 to 2.0 (NanoDrop 2000, Thermo Scientific, USA). 1ug of total RNA was reverse-transcribed for cDNA synthesis using a mix containing Superscript III reverse transcriptase (200U, Invitrogen, Carlsbad, CA, USA), DTT (10 nM), dNTP (10 mM each) and random primers (150 ng) in a final reaction volume of 20μL. Real time PCR reactions containing 5ug cDNA, ultrapure water, SyBr Green mix (ThermoFisher Scientific, Waltham, MA, USA) and specificintron-spanning primers were performed using 7500 real time PCR system (ThermoFisher Scientific, Waltham, MA, USA) (Table S1, supplementary appendix). and the following cycling parameters: 95 °C for 10 min followed by 40 cycles of 95 °C for 15 s, annealing at 60 °C for 30 s and extension at 72 °C for 30 s. The studied genes presented similar amplification efficiency (E) and the dissociation curves presented only one peak, with the respective single Tm (data not shown). Relative gene expression was calculated by the 2-DCt method [23] using the geometric mean of the housekeeping genes, that presented no alteration among groups, as the normalization factor.

### Statistical analysis

The number of animals for each experimental group was calculated by the equation n = 1+[2C(s/d)2] where “s” is the standard deviation, “d” is the difference to be detected and C is a constant calculated from C = (zα + zβ)2. By considering 90% as a power test, *p* value as 0.05 we will have “C” value is 10.51. Considering s = 0.2 and d = 0.5 we rewrite the equation with the numbers, and we have n = 1 + [2 * 10.51 * (0.2/0.5)2]; e.g. n = 4.36. We also increased the “n” value in 25% whenever it was possible to diminish sample losses due to surgical and experimental procedures during the follow-up.18F-FDG PET data were analyzed by ANOVA, using the software Prism (GraphPad Software, La Jolla California USA).

## Results

**Fig. 2:**
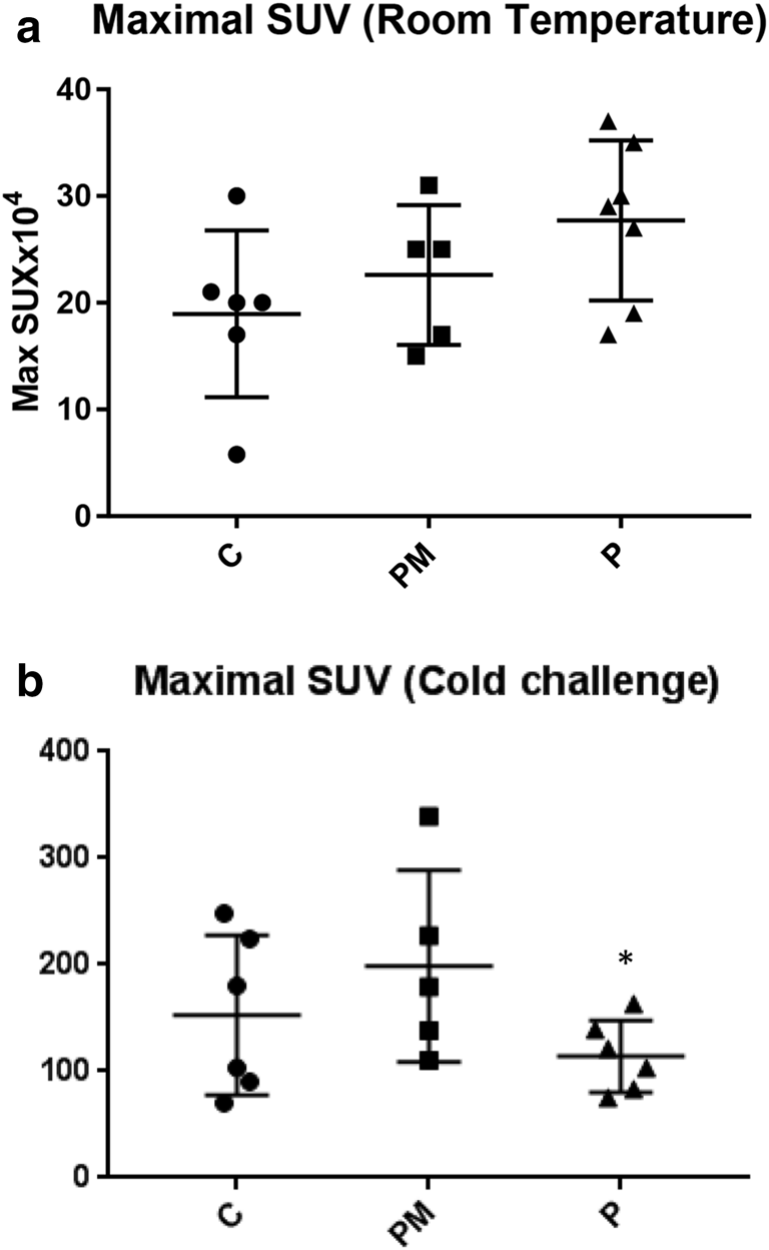
A Maximal SUV at room temperature, ANOVA test C: controls, PM: PINX Melatonin, P: PINX p = 0.13. B Maximal SUV after cold challenge, ANOVA C: controls,, PM: PINX Melatonin, P: PINX p = 0.16

The cold challenge experiment was useful and increased BAT Max SUV in all three animal models with statistical significance (Fig. 1, Additional file 1). In the PET-CT test performed at room temperature, there were no significant differences between the groups. Surprisingly, though, there was an absolute increase in Max SUV in the P group (Fig. 2a and Table 1).

**Table 1:** Maximal SUV (x10 4) and SD in all models

	PM	P	ANOVA p value
Max SUV (RT) × 10^4^	22.6 ± 6.54	27.7 ± 7.49	p = 0.13
Max SUV (cold) × 10^4^	198.6 ± 90.06	114 ± 33.69	p = 0.16
ATC	8.69 ± 3.87	4.34 ± 1.27	p = 0.03*

*statistically significant

On the contrary, when the cold exposure protocol (which has a higher sensitivity) was analyzed, the pattern was reversed, and the P group had the lowest mean values. However, once again, no statistical significance was observed (Fig. 2b and Table 1).

**Fig. 3:**
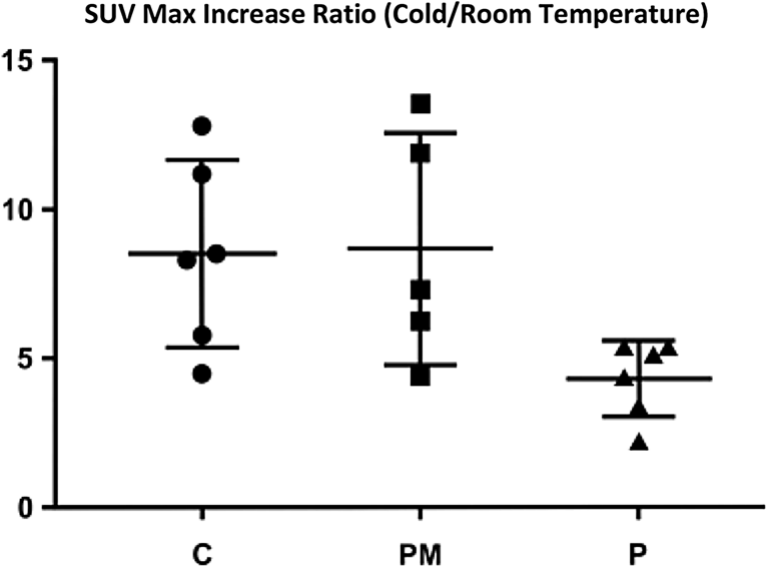
Maximal SUV increase (Cold challenge/Room temperature), ANOVA C: controls, PM: PINX Melatonin, P: PINX p = 0.0374 *Statistical significant

When the ratio of increase between the after cold challenge was compared with room temperature (what we called “acute thermogenic capacity”—ATC), we found that the P group had a statistically significant reduction in this parameter (Fig. 3 and Table 1), compared with both of the other groups. This suggests, as expected, that melatonin deficiency could impair BAT thermogenic responses (ANOVA P = 0.03), which are restored with melatonin replacement therapy.

We analyzed UCP1 mRNA in a different group of animals that received the exact same protocol.

**Fig. 4:**
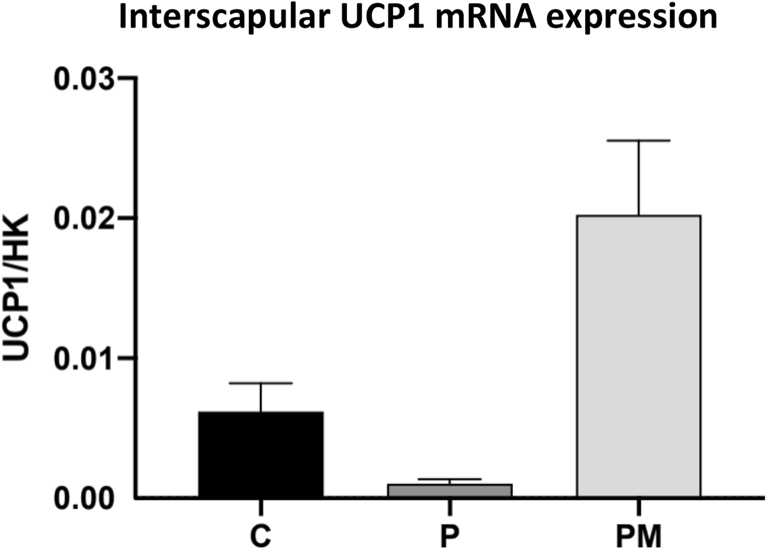
UCP1 RNA expression. ANOVA test: p < 0.001 C: controls, PM: PINX Melatonin, P: PINX

UCP1 mRNA was analyzed after sacrifice in a similar group of animals, in which the protocol conditions of C, P, and PM were identical to the animals that had the PET-CT performed. ANOVA testing revealed a P < 0.001, confirming differences between the three groups, as expected; the P group had a minimal expression of UCP1, compared with C and PM (Fig. 4). The results confirmed that melatonin deficiency reduces UCP-1 gene expression and that exogenous melatonin supplementation can reestablish its expression.

## Discussion

We aimed to test if melatonin deficiency (due to surgical pinealectomy) decreased BAT activation, and if melatonin replacement could revert this pattern to normal in Wistar rats, using the gold standard for BAT detection in humans—^18^F-FDG PET.

This is the first investigation, to our knowledge, to study BAT activation by ^18^F-FDG PET scan before and after melatonin treatment in animals. Despite data of increased BAT mass, UCP1 expression, and thermogenesis after melatonin replacement in many animal models and species already being demonstrated [3, 6, 14, 15], we found it relevant to investigate if ^18^F-FDG PET was able to detect these differences, facilitating future research in animals and humans, in which indirect measures of BAT are more feasible to be detected than through direct sample tissues. We believe that testing potential BAT activating compounds by performing an ^18^F-FDG PET in animals could help the translation of animal to human data. If animals increase their BAT SUV response to a specific compound, future studies can be done in humans to evaluate the same compound using the same method of detection.

In both isolated models (room temperature and after cold challenge), there were no differences between the groups, although some trends could be observed, including an unexpected higher maximal SUV in pinealectomized animals at room temperature and, on the contrary, a reduced maximal SUV in the P group after cold exposure. Since no statistical significance was achieved in any of the isolated methods, we prefer not to over interpret the reasons of this discrepancy. We should bear in mind that performing an ^18^F-FDG PET at room temperature is less sensitive to detecting differences; since the animals are chronically adapted to that temperature and have a small biological need to activate a recruited BAT. Perhaps in 23 °C room temperature chronic exposure, which is not thermoneutral for rats, but clearly not as challenging as an acute 4 °C exposure, other mechanisms of temperature regulation could be enough to maintain the normal thermoregulatory responses of the animal [3]. Therefore, the absence of differences between the groups in this specific model was not unexpected, at first. There has been a lot of discussion about what the ideal temperature is to perform BAT and other metabolic studies in rodents, and this remains an open question [24, 25]. The initial goal of performing the test at room temperature was exactly to observe if different patterns occurred when compared to the cold challenge and to calculate the ATC. A larger *n* could probably bring us more answers in future studies. As very few studies have analyzed BAT by PET in rats, our sample size calculation was based on a pilot study, and due to interindividual variances in Maximal SUV, we believe a larger sample size could have led us to results that were more conclusive.

However, comparing the actual ratio of BAT Maximal SUV increase, the acute thermogenic capacity (ATC), was also a primary goal, since it reveals if BAT is recruited to rapidly exert its effects on thermogenesis. In this context, the melatonin deficient group had a clearly reduced ATC, as expected. Since the PM and C groups had similar responses, this further confirms the hypothesis that melatonin proficiency is critical for thermoregulation in settings of acute cold challenging.

Indeed, the findings presented here encouraged our group to conduct an already published human study that demonstrated an increase in BAT volume and activity after melatonin replacement in a group of pinealectomized individuals [26]. Interestingly, in this study, some individuals have a reasonably high baseline BAT activity before melatonin replacement; suggesting that even if melatonin is important for thermoregulation, other mechanisms still exist.

The UCP-1 RNA data in the present study was performed as an additional tool to analyze and interpret our image data. Importantly, other published manuscripts, some of them by our own group, have already shown a decrease in UCP-1 RNA and UCP-1 protein expression after pinealectomy and a reversal of this reduction by melatonin replacement in slightly different experimental models [5, 6, 14, 15]. Therefore, regardless that our UCP-1 results are not original, they reinforce and validate the previous results and help in the interpretation of the image data [5, 6, 14, 15].

The observed pattern of UCP-1 expression in our model is in line with ^18^F-FDG PET results. The groups were statistically different; the P group has the lowest UCP-1 expression, compared with the other two groups, which in some ways could explain the lower ATC.

However, as already pointed out, an increase in UCP-1 RNA does not necessarily mean more heat production, as this recruited tissue can be inactive, if there is no need to increase thermogenesis [3, 16, 17]. Even differences in RNA and protein expression could arise, as post-transcriptional factors may influence protein synthesis. This could explain why the pinealectomized group showed a normal BAT response in ^18^F-FDG PET at room temperature, even with having lower UCP-1 expression. We did not expect differences in UCP1 expression between the C and PM groups, since our intent was to reproduce, immediately after pinealectomy, in the PM group, the physiological production of melatonin. Nevertheless, clear differences were observed, so the main hypothesis is that the replacement protocol did not exactly mimic melatonin production. As the animals drink water at specific times of the night, they would probably have several small melatonin peaks, instead of the physiological curve of night melatonin production. In addition, since the melatonin solution was only available from the start of the dark period, the duration of its plasmatic profile might reflect the winter type of melatonin secretion in some animals, changing the physiological pattern of BAT recruitment. These different kinetic curves could explain the differences observed in Fig. 4.

Our main focus in this particular study was to evaluate BAT responses in ^18^F-FDG PET. As many other different studies have demonstrated the physiological role of melatonin in body weight regulation, food intake, energy expenditure, metabolic risk factors, and many other parameters, we did not include these data as they would be redundant. Our group has previously shown that pinealectomy leads to a metabolic syndrome phenotype in rats and melatonin replacement reverts it [1]. Melatonin has been shown to decrease body weight with a minimal decrease in food intake, suggesting an effect in energy expenditure, proposed to be mediated by BAT [2, 3]. For a more comprehensive review of metabolic consequences of melatonin deficiency and the physiological role of melatonin in several animal models, see references [1, 2].

The research presented here aggregate data on the role of melatonin on thermoregulation, but the model has several limitations. Many questions still persist. Is there a role of melatonin in maintaining thermoneutrality in animals at room temperature, or is it only important if the temperature acutely decreases? Would the same results appear if the cold challenge was less intense and had a longer duration, probably a more physiological challenge? Would ATC be a reliable way to detect recruited BAT in vivo, as our study suggests? We believe so, but to detect clear effects of different compounds, we need to compare differences between the room temperature and cold challenge, since many adaptive mechanisms should exist, as thermoneutrality is vital for survival. Performing exams in only one of these conditions could lead to misinterpretations of the role of BAT and possible recruiters on thermogenesis and there is clear evidence that small differences in laboratory temperatures could lead to very different results in different animal models [24, 25, 27–29]. For example, if the tests were performed only after the cold challenge, we could conclude that no differences were seen between the groups, despite large differences in UCP-1mRNA expression. This could have led to an interpretation that UCP-1 is expressed but not necessarily translated, or that FDG-PET is not a reliable way to detect differences in BAT activation in animals.

BAT physiology is very complex, and potential compounds aiming to increase BAT can act in recruitment, activation, or both [3, 7, 11, 12]. Experimental models can help us distinguish between the action of potential BAT recruiters, such as melatonin, and the detection of BAT in vivo, by imaging techniques. Different models have the potential to investigate the role of several recruiters in different laboratory conditions, paving the way for human studies with potential compounds capable of activating or recruiting BAT, besides melatonin [11, 30]. Our finding that melatonin seems critical for acute BAT activation, induced by cold and measured by ^18^F-FDG PET, is novel. It can lead to future imaging studies in both animals and humans that will help understand the physiological role of melatonin, concerning BAT, and if melatonin could be a potential target for increasing melatonin recruitment and activation in humans, with potential therapeutic use in metabolic diseases [26]. In the same way, the finding of reduced melatonin production, leading to reduced BAT thermogenic responses, can help the understanding of increased light-at-night exposure as a potential risk factor for obesity and metabolic diseases, as already suggested [31, 32].

## Conclusions

This is the first study, to our knowledge, evaluating BAT responses to melatonin by 18F-FDG PET, which is an important essential for future melatonin and circadian studies, in animals and humans, to evaluate BAT responses through non-invasive techniques.

We demonstrated that pinealectomy impairs BAT acute thermogenic capacity, measured by ^18^F-FDG PET in Wistar rats, after an acute cold challenge, and that melatonin replacement reverses this impairment, in line with the results obtained by UCP1 RNA expression. However, when the results at room temperature and after the cold challenge were analyzed separately, no significant differences were seen, highlighting the complexity of thermoregulation and the possible limitations of ^18^F-FDG PET to detect differences in BAT, depending of the temperature conditions.

These results corroborate many experimental models that suggest that melatonin has a critical role in BAT mass and activity. Studying different compounds that potentially increase BAT by an imaging technique considered to be the gold standard in humans could facilitate future studies in our species.

## Supplementary information

**Additional file 1: Table S1.** Primers used for RT-PCR for UCP-1 expression. **Figure S1.** Maximal SUV (x10^4^) in all experimental groups in room temperature and after cold exposure.

## Data Availability

The data and materials are available, if needed.
