# Supplementary material for: Melatonin deficiency decreases brown adipose tissue acute thermogenic capacity of in rats measured by 18F-FDG PET
Source: Diabetol Metab Syndr. 2020 Sep 21;12:82. doi: 10.1186/s13098-020-00589-1 (PMC7504678; doi:10.1186/s13098-020-00589-1)
Supplement: Supplementary file 1 — Additional file 1: Table S1. Primers used for RT-PCR for UCP-1 expression. Figure S1. Maximal SUV (x104) in all experimental groups in room temperature and after cold exposure. [file 13098_2020_589_MOESM1_ESM.docx]

Additional appendix

Table S1 – Primers used for RT-PCR for UCP-1 expression

| **Primer** | **Acess Number** | **Sequence (5’-3’)** | **Fragment** | **Location** |
| --- | --- | --- | --- | --- |
| Uncouplin Protein 1 (Ucp1) | NM_012682 2 | GCCAAGACAGAAGGATTGCC  CAGCCGAGATCTTGCTTCCC | 163bp | 377-539 |
| Beta actin (Actb) | NM_031144 | CTAGGAGCCAGGGCAGTAATCT  AAGACCTCTATGCCAACACAGTG | 97bp | 117-214 |
| Ribosomal protein L37a (Rpl37a) | NM_001108801 | CGCTAAGTACACTTGCTCCTTCTG  GCCACTGTTTTCATGCAGGAAC | 93bp | 161-254 |

Figure S1 – Maximal SUV (x10 ^4^) in all experimental groups in room temperature and after cold exposure


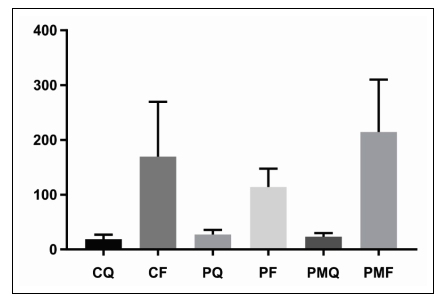


CQ- Controls in room temperature

CF –Controls in cold

P- PINX in room temperature

PF – PINX in cold

PMQ –PINX supplemented with melatonin in room temperature

PMF – PINX supplemented with melatonin in cold

Paired t test:

CQ versus CF – p=0.00142

P versus PF – p=0.0128

PMQ versus PMF –p=0.0284
